# Bursting Rate Variability

**DOI:** 10.3389/fphys.2021.724027

**Published:** 2021-12-02

**Authors:** Roberto Martin del Campo Vera, Edmond Jonckheere

**Affiliations:** ^1^Department of Neurological Surgery, University of Southern California Keck School of Medicine, Los Angeles, CA, United States; ^2^Department of Electrical and Computer Engineering, University of Southern California, Los Angeles, CA, United States

**Keywords:** Heart Rate Variability, electromyogram, bursting activity, Akaike and Bayes information criteria, return time distribution, Daubechies, doublets

## Abstract

In this paper, a new electromyographic phenomenon, referred to as *Bursting Rate Variability (BRV)*, is reported. Not only does it manifest itself visually as a train of short periods of accrued surface electromyographic (sEMG) activity in the traces, but it has a deeper underpinning because the sEMG bursts are synchronous with wavelet packets in the D8 subband of the Daubechies 3 (db3) wavelet decomposition of the raw signal referred to as “*D8 doublets”*—which are absent during muscle relaxation. Moreover, the db3 wavelet decomposition reconstructs the *entire* sEMG bursts with *two* contiguous relatively high detail coefficients at level 8, suggesting a high incidence of two consecutive neuronal discharges. Most importantly, the timing between successive bursts shows some variability, hence the BRV acronym. Contrary to *Heart Rate Variability (HRV)*, where the R-wave is easily identified, here, time-localization of the burst requires a statistical waveform matching between the “*D8 doublet”* and the burst in the raw sEMG signal. Furthermore, statistical fitting of the empirical distribution of return times shows a striking difference between control and quadriplegic subjects. Finally, the BRV rate appears to be within 60–88 bursts per minute on average among 9 human subjects, suggesting a possible connection between BRV and HRV.

## 1. Introduction

Under some conditions, the surface electromyographic (sEMG) activity recorded along the paraspinal muscles of human subjects shows some standing wave properties, even though the trunk does not manifest a visually obvious movement (Jonckheere et al., 2010; Martin del Campo and Jonckheere, 2016). Such conditions can be reproduced by putting the research subject in the prone position and applying light pressure at some specific “gateway” points of the spine (usually the neck and the coccyx) to elicit the oscillation.

The motion usually starts in a chaotic fashion at the distal ends of the spine, propagates caudally, until it settles in a standing wave pattern, which can undergo “period halving bifurcations,” transitioning away from chaos (Martin del Campo and Jonckheere, 2017). At that stage, no further digital stimulus is required, indicating that the rhythmic movement is innervated by a Central Pattern Generator (CPG) (Marder and Calabrese, 1996; Marder and Bucher, 2001), as argued in (Jonckheere et al., 2010). A further confirmation of the CPG hypothesis is that two quadriplegic subjects have been able to sustain the so-called *spinal wave* (Jonckheere and Lohsoonthorn, 2004; Jonckheere et al., 2010; Musuvathy and Jonckheere, 2010).

Besides CPG, another important aspect of this movement is *coherence at a distance*; the antinodes of the wave are indeed in coherent motion, with a wavelength in the order of ~1 m, hence qualifying as *coherence at a distance* (Farmer, 1998; Farmer et al., 1998; Kopell, 2000).

The specificity of this CPG-innervated, coherent motion is confirmed by the Daubechies 3 wavelet decomposition of the sEMG signal, more specifically, by wavelet packets repeating themselves in an aperiodic fashion in the D8 subband of the wavelet decomposition, referred to as “*D8 doublets”* (Mallat, 1989; Daubechies, 1992).

The appearance of this waveform in both control and quadriplegic subjects during the spinal wave phenomenon, as well as its absence when the spinal wave is not sustained, has suggested that the d8 doublets are a type of biological marker specific to this phenomenon. This further motivated the inclusion of quadriplegic patients in the pool of research subjects to confirm that their depleted neuro-skeletal system impacts the doublet return time.

**Figure 1:**
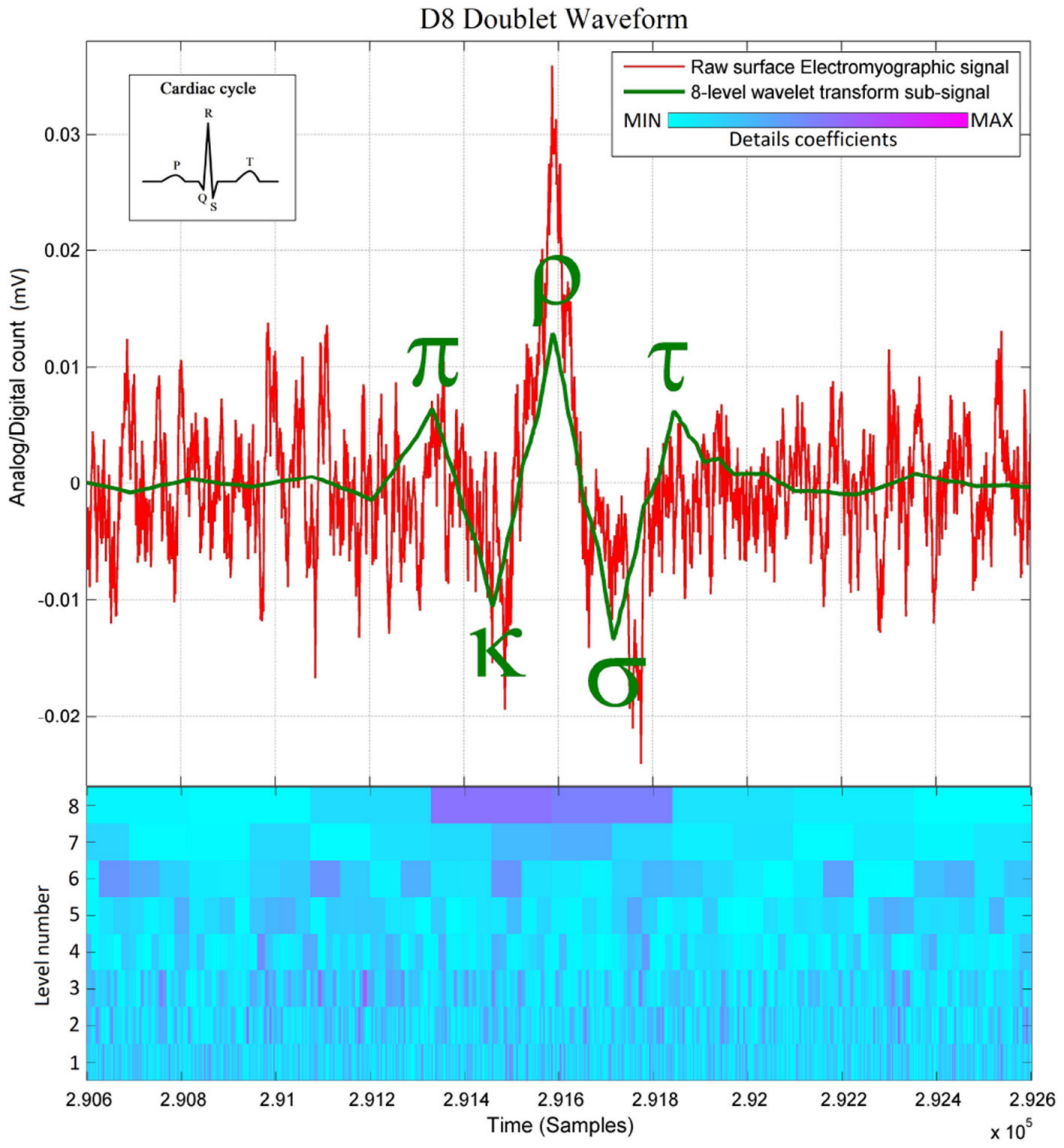
Raw paraspinal surface electromyographic signal of a healthy individual, overlaid with its 8-level wavelet transformed sub-signal and its scalogram below *(The level number in the scalogram corresponds to the scale of the discrete wavelet transform dwt of Matlab)*. Note that the D8 doublet is a precise sequence of (+) peaks and (−) dips, defined here as the π-κ-ρ-σ-τ sequence because of its similarity with the cardiac cycle. The concordance between the D8 and the burst here appears naturally without preprocessing. Observe the two D8 coefficients indicating rapid succession of 2 MUAPs.

Since these *D8 doublets* are reconstructed from two relatively high details coefficients at level 8 (Figure 1), and due to the surface electromyography being a superposition of multiple Motor Unit Action Potential (MUAP) trains (see Cram et al., 1998, Figures 3, 4), it is still unclear at this stage whether the observed D8 doublets are double discharges of the same motor units (see Bawa and Calancie, 1983; Piotrkiewicz et al., 2013; Mlrowczynski et al., 2015) for further details on the double discharge phenomenon), or single discharges of two motor units firing rhythmically one after the other. In either case, the observed D8 doublet waveforms represent a *highly synchronized rhythmic spiking phenomenon of multiple neurons* that would conform to the definition of an “*exceptional doublet”* of Piotrkiewicz et al. (2013, Figure 5B) if two firings are coming from the same motor unit, “exceptional” in the sense of large intradoublet interspike interval. Indeed, the interspike period of ~60 ms—here measured peak-to-peak from the D8 doublet waveform—exceeds the conventional limits of 2-20 ms according to the standardization of doublets set by the American Association of Neuromuscular & Electrodiagnostic Medicine (The AANEM Nomenclature Committee, 2015). However, it has been reported in (Piotrkiewicz et al., 2013) that the standard range of doublets can be exceeded as Piotrkiewicz et al. (Piotrkiewicz et al., 2013) report exceptional doublets with 37 ms of intradoublet time in the human soleus muscle. Furthermore, the Discrete Wavelet Transform (DWT) has also served to obtain the entire spiking event duration of ~130 ms measured from onset to offset of this wavelet waveform at scale 8, which spans the time comprised by two relatively high details coefficients at this scale (Rodriguez-Carreño et al., 2012, Figure 11).

**Figure 2:**
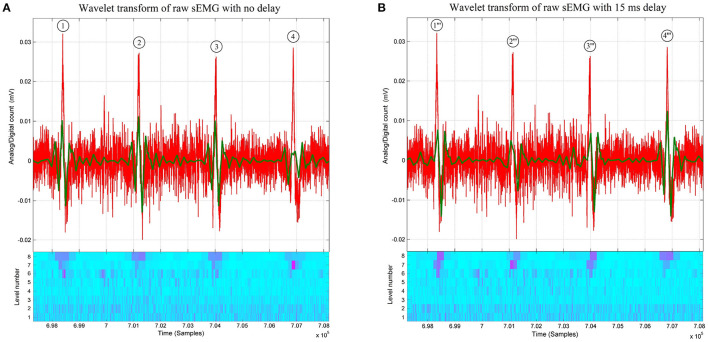
**(A)** Raw thoracic surface electromyographic signal (in red) of control subject #4, overlaid with its 8-level wavelet transformed sub-signal (in green) and its scalogram below *(Conventions are the same as in* *Figure 1**)*. Note that at the first, second, and third bursts the matching between the raw sEMG signal and its 8-level sub-signal is better than the one at the fourth burst. The recovery of the matching between the fourth burst and its D8 doublet is shown in Figure 3, where different time delays are applied before wavelet processing. **(B)** Raw thoracic surface electromyographic signal (in red) of subject #4 (control) time-shifted by 15 ms (in red), overlaid with its 8-level wavelet transformed sub-signal (in green) and its scalogram below *(Conventions are the same as in* *Figure 1**)*. A time shift of 15 ms (skipping the first 60 samples) on the raw signal before wavelet processing is sufficient for the fourth D8 doublet to better match the raw sEMG signal than when no time shift is applied to the raw signal as in **(A)**.

Besides these early findings, the crucial observation that launched this research is the near-synchrony between the onsets of the doublets observed on the D8 traces and the onsets of the bursts of accrued sEMG activity visible on the raw signal traces, as shown in Figures 1, 2A. The “nearness” of the time localizations of the doublets and the bursts is crucial here. Indeed, by definition of the wavelet decomposition, the repetition of the DWT frame generating the D8 subband is periodic, while the sequence of bursts is not. Therefore, some time-shifting of the raw signal trace is necessary to acquire a good *waveform matching* between a *specific* burst and its D8 doublet. It turns out that this time-shifting is different from one burst to the other, leading to a variability of the time interval between successive bursts, which is referred to as *Bursting Rate Variability (BRV)*.

The adopted wavelet transform technique fits within a broad range of techniques that aim at accurately localizing in time an “event,” say R-wave of ECG, by correlating the event waveform with the mother function of a specific wavelet transform. In particular, this has been exploited for time-localization of R-wave, resulting in accurate dynamical modeling of interbeat interval with application to sleep apnea (Ivanov et al., 1996; Mietus et al., 2000; Ivanov, 2001).

## 2. Methods

### 2.1. Methods: Control and Quadriplegic Subjects

For our analysis, a population of 9 human subjects, 7 control (healthy) and 2 quadriplegic subjects (presenting a total of ~8,000 *doublets*), were chosen. The quadriplegic subjects both had a cervical spinal cord injury at the C5 vertebral level (Jonckheere et al., 2010; Musuvathy and Jonckheere, 2010).

Before recordings, the subjects had signed the Informed Consent drafted by the investigators and approved by the University Park Campus (UPC) Institutional Review Board (IRB) of the University of Southern California. The subjects were recruited on the occasions of several meetings. Participation was purely voluntary, gender-neutral, age-neutral. Pool consisted of 60% females–40% males, in the 40's age group. Excluded were such vulnerable subjects as pregnant women, mentally disabled persons, prisoners, subjects with chronic illnesses, and low income workers. Included were quadriplegic patients and children, but no children participated.

The sample size of the quadriplegic subjects is very small for obvious reasons. This translates in a low burst sample size compared with control subject. Given this limitation, the best that could have been done was to rely on the robustness of the Weibull distribution, which has historically been specially devised for small sample sizes (see section 3). Admittedly, if more quadriplegic patients become available, results might have to be revised.

The fact that the recruited quadriplegic subjects were experiencing partial recovery from their spinal cord injuries provided further data on how the quantitative properties of the “doublets” relate to their neuro-skeletal function deficit as compared with control subjects. To draw an objective comparison between quadriplegic and control subjects, every quadriplegic subject had its recording taken during the same session as a control subject.

### 2.2. Methods: Electrode Placement

The data utilized in this investigation have been recorded over a period of a little more than 10 years. All along those recordings, we have followed a consistent recording protocol: Surface electromyography (sEMG) reduced-noise tripolar electrodes (“Uni Patch Tyco EMG Electrodes Round Disk 7500 2.25 diameter Ag snaps” with two inputs to a differential amplifier and one grounding electrode) were placed at cervical (C2-C3), thoracic (T4-T6), lumbar (L3), and sacral (S2-S4) positions; all with the same sampling rate of 4,000 samples per second. The sensitive input prongs of the front-end electronics were initially at a 45-deg. angle with the muscle fibers and subsequently aligned with the back-muscle fibers, without significant difference observed in the results.

### 2.3. Methods: Equipment

The most recent (<4 years) recordings were made with an Insight Discover sEMG station together with a Measurement Computing^*TM*^ USB-1608FS device for analog-to-digital conversion, while the earlier recordings (10 years ago) were done with an Insight Millennium sEMG station interfaced with a Computer Board PCMCIA DAS16/16 card analog-to-digital converter.

The consistency of the results across two experimental platforms indicates that the observed doublet phenomena are unlikely to be due to artifacts of the experimental equipment (Martin del Campo and Jonckheere, 2016, 2017).

### 2.4. Methods: Wavelet Transform

We picked up the Daubechies 3 (db3) wavelet decomposition, originally for the motivation that its D8 subband provided the best correlations among such subband of signals recorded at different points along the spine, hence promoting the “coherence at a distance” aspect (Jonckheere et al., 2010). Later, however, it was discovered that under some conditions the D7 subband was preferable (Martin del Campo and Jonckheere, 2017). Parallel to this line of thoughts, it was found by the present and other investigators that the mother function of the db3 mimics the MUAPs detected by the electrodes, which makes the DB3 the ideal tool for picking up relevant waveforms in an otherwise messy sEMG signal corrupted by noise and motion artifacts (Sloboda and Zatsiorsky, 1997; Jonckheere et al., 2010).

### 2.5. Methods: Waveform Matching

To solve the “shift variance” problem of the DWT of many signals causing in our particular application some of the D8 doublets not to have well-defined peaks and dips (see doublet #4 in Figure 2A), or causing the π*κρστ* peaks and dips to be horizontally offset with respect to the raw sEMG (see the ρ-peak of D8 doublets #1 and #3 in Figure 2A), we then shifted the sampling times of the wavelet coefficients by delaying the time at which the DWT begins (Bradley, 2003). This makes the coefficients span different sections of the same raw sEMG signal.

**Figure 3:**
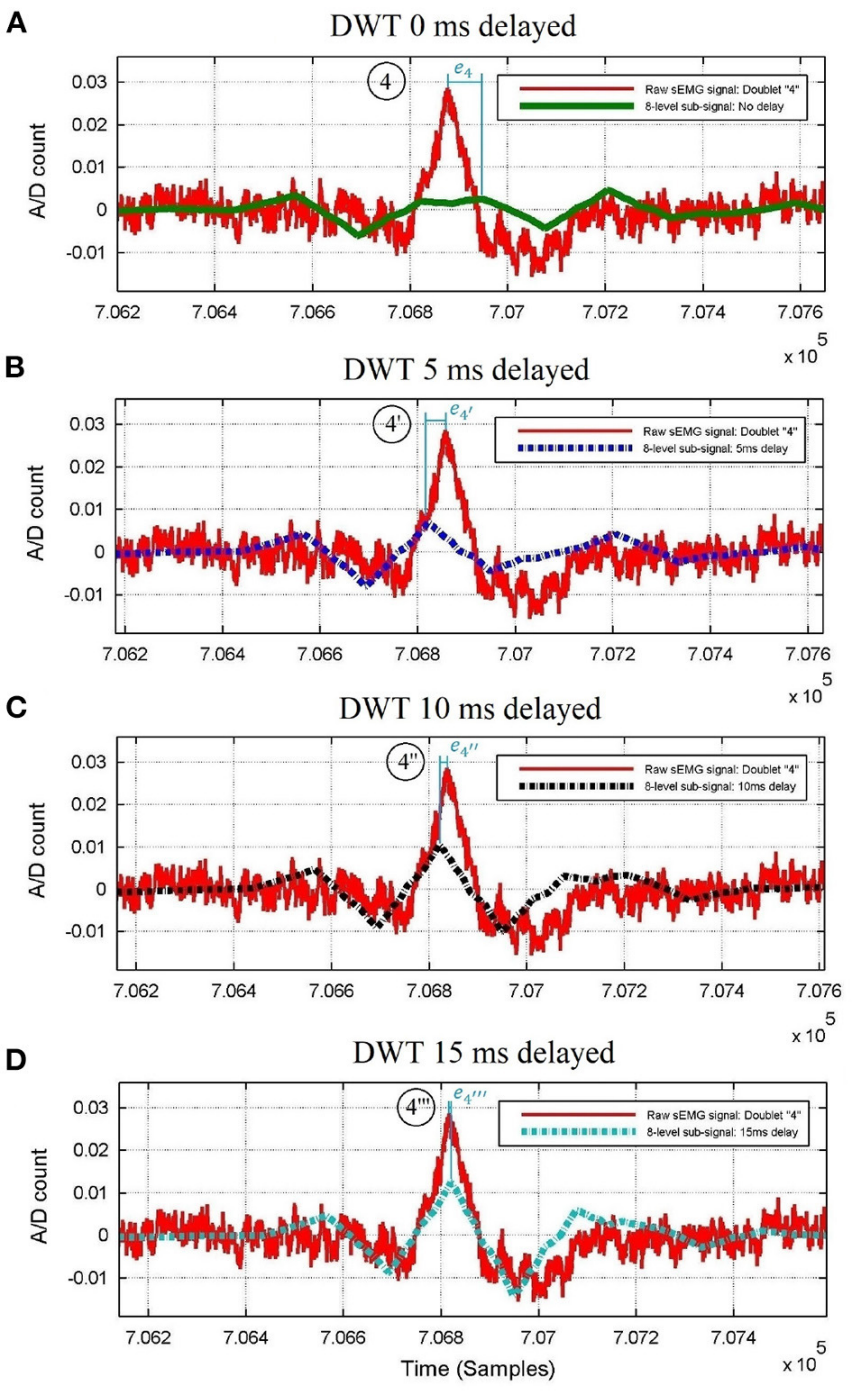
Raw thoracic surface electromyographic signal of control subject #4 centered around doublet #4 of Figure 2, superimposed with its 8-level wavelet transformed sub-signal when **(A)** no delay, **(B)** 5 ms delay, **(C)** 10 ms delay, and **(D)** 15 ms delay is applied to the raw sEMG signal before wavelet processing. The peak time errors of the ρ-wave are |*e*_4_|>$\left|e_{4^{\prime }}|>|e_{4^{\prime \prime }}|>|e_{4^{\prime \prime \prime }}\right|$; thus, the D8 doublet with the smallest error (#4”') belongs to the Pareto-optimal front, whereas doublets #4, #4', and #4” do not.

A matching increment of the π-wave, πκ-slope, κ-wave, κρ-slope, ρ-wave, ρσ-slope, σ-wave, στ-slope, and τ-wave with the raw sEMG burst around doublet #4 of Figure 2A is obtained by omitting the first 20, 40, and 60 samples (see Figures 3B–D, respectively) before wavelet processing. This results in recovering the precise peak-dip sequence of the π*κρστ* complex of doublet #4”' in the raw burst signal, until achieving the benchmark waveform match of Figure 1.

Due to the variability in the occurrence of the bursts, the process of delaying the raw sEMG signal before wavelet processing to achieve optimal waveform matching is different from one raw bursting waveform to another one. Figure 2B shows how the best-suited time delay for doublet #4”' is not the best suited for doublets #1”', #2”', #3”', as compared with doublets #1, #2, #3 of Figure 2A when no time delay had been applied.

To make the above procedure optimal, the errors of this π*κρστ* waveform vs. sEMG burst are gathered in a multi-objective optimization function, of which the Pareto front is identified using expert rules. If a given delay for the π*κρστ* waveform parameters is on the Pareto-optimal front as in Figure 3D, then this provides a good match; whereas if some delays are not on the Pareto-optimal front as those in Figures 3A–C, then they do not provide a good match.

**Figure 4:**
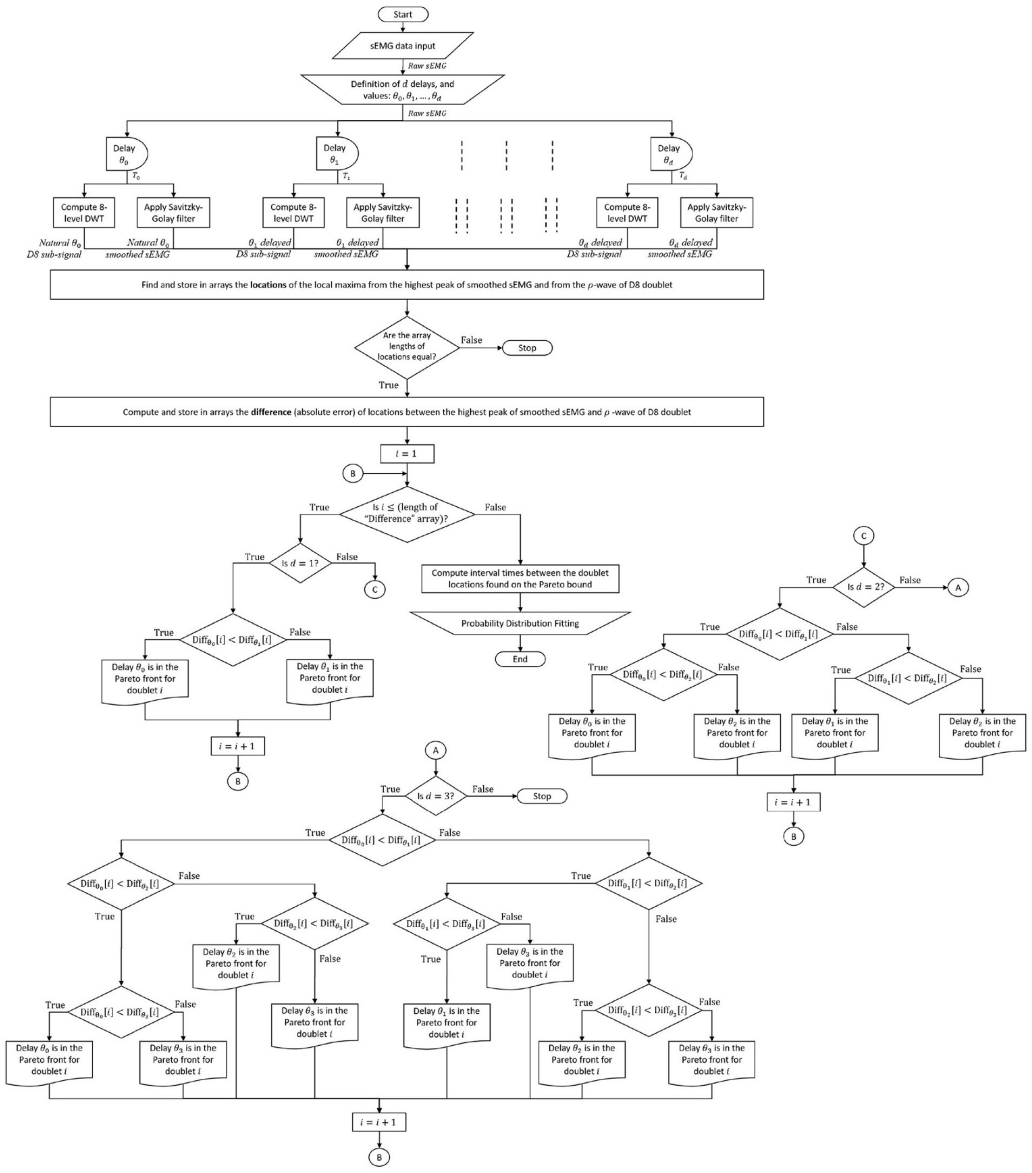
Complete sEMG signal processing and analysis flowchart: The raw sEMG signal passes through *d* predefined delays to time shift its 8-level sub-signal and its smoothed sEMG signal. In the example of this paper, the number of delays is *d* = 3, with values of θ_0_ = 0 ms, θ_1_ = 5 ms, θ_2_ = 10 ms, θ_3_ = 15 ms, a guideline for defining the delay values is less than one cycle ( ≤ 30 ms) since Figure 3 spans half cycle (15 ms). The expert system finds the minimal error between local maximum of the sEMG burst and ρ-wave of its 8-level sub-signal (shown in Figure 3) among the (*d*+1) pairs of signals that come from the same sEMG trace, enhancing waveform matching and providing the Pareto front ρρ-interval times (and/or ρ-wave magnitudes) for probability distribution analysis.

The expert system that automatically obtains the minimal error between the ρ-wave locations of each D8 doublet and its raw burst (vertical lines in Figure 3), consist of a series of nested conditional statements (*if-then-else* rules depicted in Figure 4) inside a *for* loop, with index value that identifies the chronological position of each doublet appearing in the time series.

Due to the raw sEMG signal being corrupted with high-frequency noise, and in order to increase the precision in finding the times at local maxima for each D8 doublet, a Savitzky-Golay filter is first applied to the raw sEMG before being processed by the expert system. The number “*d”* of testing delays used in the present paper to exemplify the waveform matching technique is preset to 3, where values of 5 ms, 10 ms, and 15 ms have been assigned for each delay time. It is worth noting that the expert system also considers the case of no delay time for the D8 doublets that are naturally matched with the sEMG bursts, which is represented with a zero-delay time.

The whole sEMG signal processing technique, from data collection to probability distribution fitting, passing through multi-delayed 8-level DWT, smooth filtering, and the expert rules is depicted in the flowchart of Figure 4.

### 2.6. Methods: Histogram vs. Theoretical Probability Distribution of Return Times and Statistical Software

**Figure 5:**
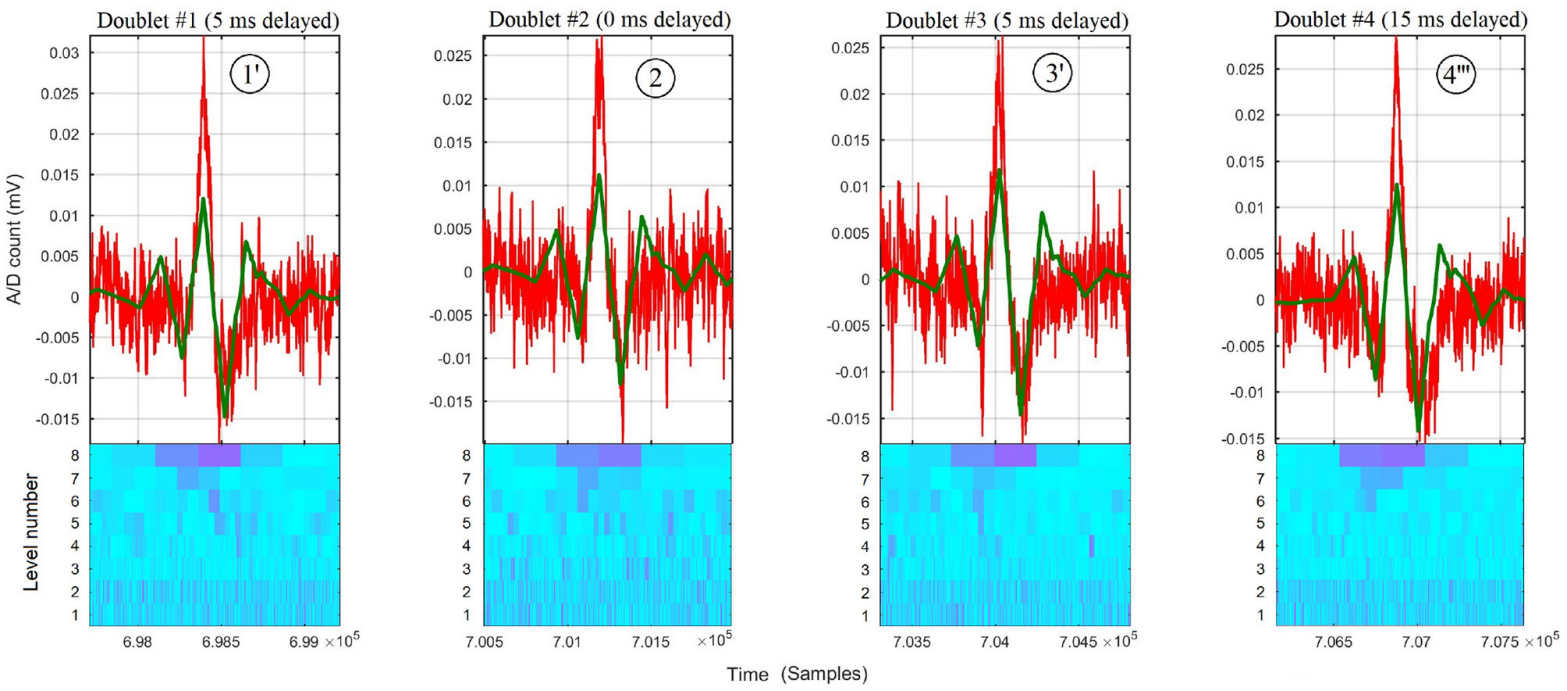
Zoom around each burst of raw thoracic sEMG trace (in red) of subject #4, overlaid with its 8-level sub-signal (in green) and its scalogram below *(Conventions are the same as in* *Figure 1**)*. The D8 doublets #1', #2, #3', and #4”' correspond to time-shifting the DWT at their respective Pareto front time delays of 5, 0, 5, and 15 ms, resp., obtained for each doublet by the expert system.

There are two ways to construct the histogram of the time between the ρ-waves of successive D8 waveforms, or “*return time”* for short. In one procedure are included in the sample set only those ρρ-intervals between ρ-waves that are *naturally* matched with their corresponding bursts (without time shifting), discarding those that do not appear to match, such as doublet #4 of Figure 2A. However, some doublets might appear to *naturally* match with their sEMG bursts—for instance, doublets #1 and #3 of Figure 2A—and considering the ρ-wave locations of these two doublets would add undesired observational systematic errors to the distribution fit analysis since a better match was found by the expert system (see doublets #1' and #3' of Figure 5). These unwanted errors can be eliminated in the other “*enhanced”* procedure achieved with the expert system and the time-shifted DWT at various time delays; thus, the latter procedure is preferred.

In the *enhanced* procedure, the histogram is constructed with accrued accuracy with ρρ-intervals after optimal (on the Pareto-optimal front) time shifting to match *all* bursts with their respective D8's. The latter, by the same token, also increases the sample size.

The statistical software SAS® Studio 3.4 and JMP Pro 13 (both by the SAS Institute) were used to find the best theoretical probability distribution fit from the frequency histograms of doublet return times based on the (corrected) Akaike Information Criterion (AICc) for model selection; the results are summarized in Tables A1–A3 in Appendix. In the case where the Weibull distribution was the best ranked in the AICc sense, it was checked for a goodness of fit using the Cramer-von Mises-W test. As for the best-ranked normal mixture distribution, the Pearson's chi-squared test was used. In both cases, the *null hypothesis* (*H*_0_) states that the observed frequency distribution is consistent with the estimated theoretical distribution, and small *p*-values (<0.05) would reject *H*_0_ in favor of the *alternative hypothesis* (*H*_1_) that the data is not from the theoretical distribution.

### 2.7. Methods: Model Selection Criterion and Sample Size Guidelines

#### 2.7.1. Best Fitting Probability Distribution

Akaike's approach to finding the best probability distribution fit is a Maximum Likelihood Estimation technique that seeks to provide a measure of fitting relative to distinct probability models by estimating parameters that maximize their Likelihood function (Akaike, 1974).

The corrected Akaike Information Criterion (correction for overfitting), is defined as $AICc=AIC+\frac{2k\left(k+1\right)}{n-k-1}$, where *n* is the sample size, *k* is the number of parameters, and *AIC* = 2*k*−2*LogLikelihood*(θ), where θ represents the parameters to be estimated for a given model.

Let *X*_1_, *X*_2_, …, *X*_*n*_ be a set of continuous random samples with joint density function *f*_θ_(*X*) depending on the parameters θ. The *Likelihood* function *L*(θ) = *f*_θ_(*x*_1_, *x*_2_, …, *x*_*n*_), sometimes written as *L*(θ|*x*), is the joint probability distribution *f*_θ_(*x*_1_, *x*_2_, …, *x*_*n*_) with parameters θ of the set of *n* random variables evaluated at the observed values from the sample. In practice, the *Likelihood* function factors as *f*_θ_(*x*_1_)*f*_θ_(*x*_2_)…*f*_θ_(*x*_*n*_). The *LogLikelihood* represents the natural log of the *Likelihood* function, which is often preferred as it simplifies the calculations of critical values.

Since there is no prior knowledge of the underlying distribution of D8 doublet return times, the AICc—by means of estimating the parameters that provide the largest plausibility for obtaining the observed values for several probability models—provides a point of comparison among probability models that the samples are most likely to come from, serving as a means for model selection. Some of the models tested face-to-face in this sense include the Gamma, Weibull, Exponential, LogNormal, GLog, Johnson Su, Johnson Sl, Gaussian, and Normal 2 & 3 Mixture probability densities.

The Akaike Information Criterion reformulates the maximization of the *LogLikelihood* function by working with its negative value (minimization of the *LogLikelihood* function), in such case, lower values of AICc denote better model fits (Akaike, 1974).

Since the AICc only provides a ranking among different types of distributions and does not warn for poorly fitted models, a Goodness-of-fit test for the model with the lowest AICc complements this part of the model selection technique, ensuring that the best-ranked model represents a good fit.

#### 2.7.2. Sample Size Guidelines

To construct guidelines on the minimum and the maximum number of return times to consider in the distribution fit analysis, we performed simulations (with 5,000 trials at different sample sizes in the range from 5 to 5,000) by random sampling from an underlying distribution and obtained the number of times a given distribution was the best fit in the AICc sense.

The two-parameter Weibull distribution (α, β)


$$f\left(x;\alpha ,\beta \right)=\frac{\beta }{\alpha }{\left(\frac{x}{\alpha }\right)}^{\beta -1}e^{-{\left(\frac{x}{\alpha }\right)}^{\beta }};\;for\;\alpha ,\beta >0;x\geq 0,$$

where α and β are the scale and shape parameters, respectively, was found to be the most robust at small sample sizes as it required the smallest sample size (*n*) to be identified as the best fit most of the trials (Figure A1 in Appendix). For instance, at least *n*≈6 samples were required to achieve ~50% success rate for several values of α and β, compared with Gamma (λ = 4, *scale* = 1) with at least *n*≈26 with ~32% success rate, Gaussian (μ = 100, σ^2^ = 30^2^) with at least *n*≈130 with ~40% success rate, among others shown in Figure A2 in Appendix. Due to the high robustness of the Weibull distribution at small sample sizes that we observed with simulations, it is not surprising that the Weibull distribution is widely applied in reliability tests, often hampered with small sample sizes (Hisada and Arizino, 2002; Lu and Wang, 2008; Jiang et al., 2015).

In regard to the maximum number of return times to consider in the distribution fitting analysis, a stopping rule can be determined when the AICc approaches a minimum value, meaning that the percentage change of AICc approaches zero as the sample size increases (Figures A3, A4 in Appendix).

## 3. Results

### 3.1. Results: Waveform Matching

Although the time localization in the first three doublets of Figure 2A were lost in the process to retrieve the ρ-wave of D8 doublet #4, as shown in Figure 2B, the expert system recovered and found the Pareto time localization of the other doublets at each predefined delay.

With the predefined values used to exemplify the waveform matching technique (0, 5, 10, and 15 ms), the delays θ_1_ = 5 ms for doublet #1', θ_0_ = 0 ms for doublet #2, θ_1_ = 5 ms for doublet #3', and θ_3_ = 15 ms for doublet #4”' have been found by the expert system to be on the Pareto front (see Figure 5).

### 3.2. Results: Theoretical Probability Distributions

**Figure 6:**
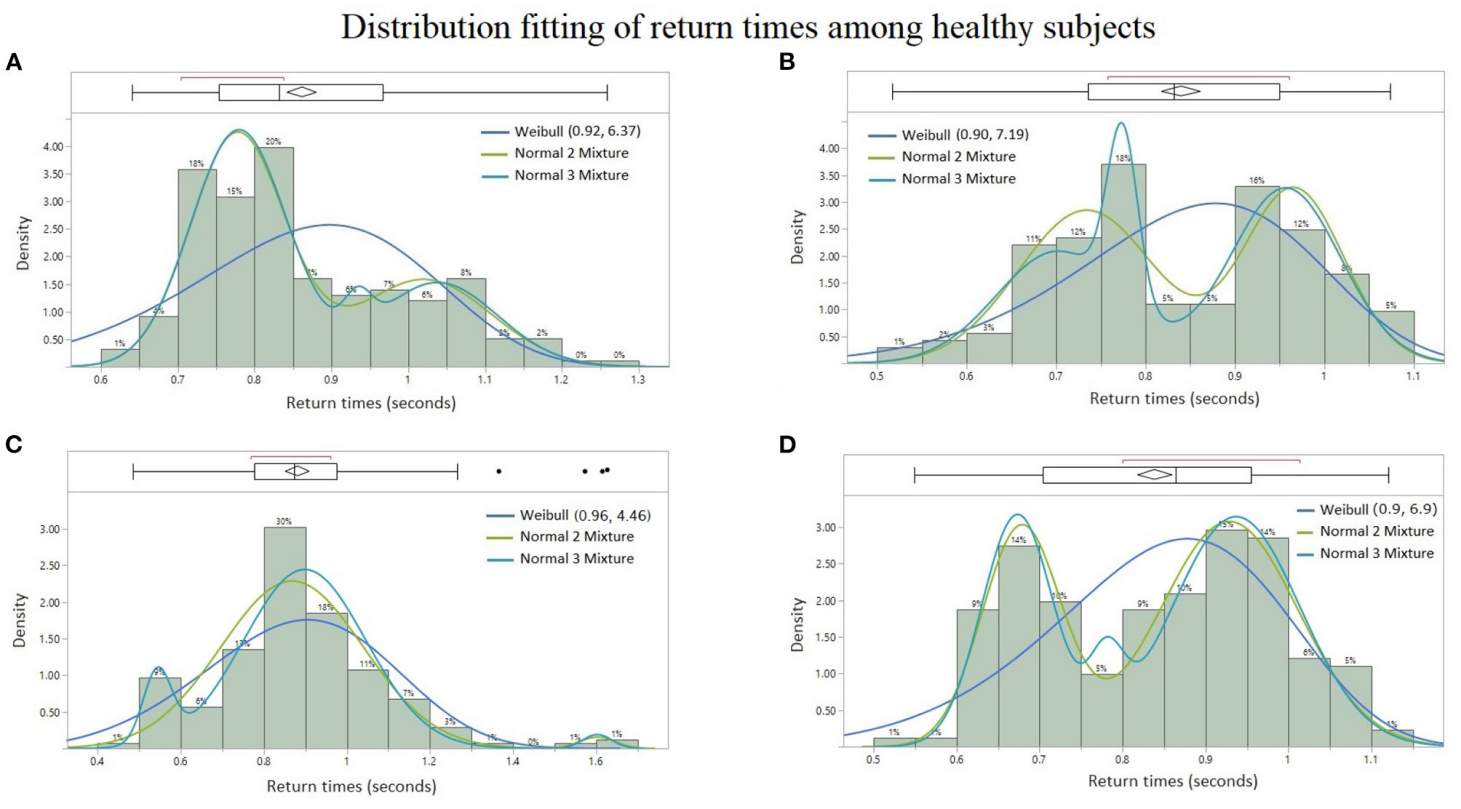
Probability distribution fitting of return times from healthy subjects: **(A)** lumbar spine signal of Subject #6 with Normal 2 Mixture (*AICc* = −272.53), Normal 3 Mixture (*AICc* = −265.98), and Weibull (*AICc* = −199.41), **(B)** sacral signal of Subject #8 with Normal 3 Mixture (*AICc* = −187.61), Normal 2 Mixture (*AICc* = −186.31), and Weibull (*AICc* = −167.78), **(C)** cervical signal of Subject #5 with Normal 3 Mixture (*AICc* = −95.26), Normal 2 Mixture (*AICc* = −87.19), and Weibull (*AICc* = −51.56), and **(D)** cervical signal of Subject #8 with Normal 2 Mixture (*AICc* = −236.16), Normal 3 Mixture (*AICc* = −230.59), and Weibull (*AICc* = −194.86). Lower AICc values indicate a better distribution fit.

**Figure 7:**
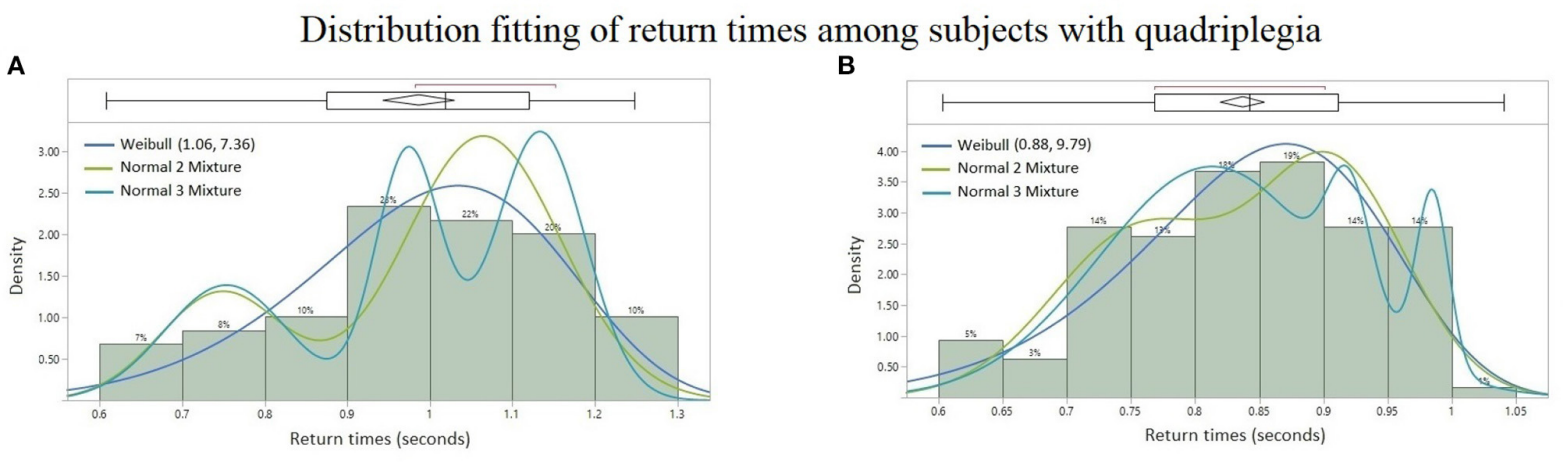
Probability distribution fitting of return times from patients with quadriplegia: **(A)** Thoracic signal of Subject #2 with Weibull (*AICc* = −45.69), Normal 2 Mixture (*AICc* = −41.36), and Normal 3 Mixture (*AICc* = −35.46), **(B)** lumbar spine signal of Subject #1 with Weibull (*AICc* = −231.43), Normal 2 Mixture (*AICc* = −227.36), and Normal 3 Mixture (*AICc* = −227.12). Lower AICc values indicate a better distribution fit.

Among all the paraspinal signals, the Weibull distribution was found to be the best probability fit in the AICc sense among the quadriplegic subjects as shown in Figures 7A,B, whereas the Normal 2 & 3 Mixtures were prevalently the best fit among control subjects as shown in Figures 6A–D. The parameter estimates for the best-fitted distributions are reported in Tables A1–A3 (Appendix).

### 3.3. Results: Control vs. Quadriplegic Subjects

The contrasting difference in the results of the present study between quadriplegic and control patients, namely in their probability distributions of doublet return times and sample sizes, points to “doublets” becoming more prevalent (and with multimodal return times) in healthy neuromuscular systems than unhealthy ones. Furthermore, the more predominant rhythmic synchronization of neurophsyological activity of healthy subjects is consistent with the hypothesis that coherence at a distance is an indication of the nervous system able to coordinate the activity of many muscles (Farmer, 1998; Farmer et al., 1998; Jonckheere et al., 2010).

More specifically, the empirical distribution of the time intervals between successive bursts differ from one subject to another, but the types of continuous probability distributions already differentiates the BRV of quadriplegic (Figures 7A,B) vs. control subjects (Figures 6A–D). The quadriplegic subjects consistently presented the *Weibull* distribution as the best fit, and *mixtures of normal* distributions in the case of control subjects; this discrepancy already points to some neurophysiological applications of BRV. Note that the Weibull distribution is known as a Type III *Extreme Value* distribution (Faranda et al., 2012).

## 4. Discussion

### 4.1. Daubechies 3 (db3) vs. Other Wavelet Mother Functions

In earlier studies, a wavelet similar to db3, the Daubechies 4 (db4), did not provide satisfactory results as it failed to pick up the onset of some bursts as accurately as the db3 did it (Jonckheere et al., 2010; Martin del Campo and Jonckheere, 2016, 2017). Additionally, the db3 mother function adhered more to the “pointy” shape of observed crests and troughs of electromyographic bursts than the smoother db4 mother function. Another wavelet tested face-to-face against db3, the Daubechies 2 (db2), did not show clear boundaries between the bursts start and end points, compared to the db3. Finally, it was also found that the D8 of the db3 allowed for accurate localization of sEMG bursts most of the times.

### 4.2. Neurophysiological Personality

In the present study, we hypothesize that the rhythmic bursts represent a synchronization of multiple MUAPs firing exceptional doublets, and that there is a probable connection between them and the dynamical system theory of the return time of rare events (Haydn et al., 2005, 2014), the Generalized Extreme Value (GEV) theory of such rare events (Freitas et al., 2010; Freitas et al., 2011) and the neurophysiological studies by Piotrkiewicz et al. (2013). In the last-mentioned studies, double-firing motor units classified as single, repetitive, and exceptional doublets, constituted a small percentage (9.5%) of recorded neuronal discharges and were considered as “unusual” discharges, whereas the exceptional type was even more unusual (~1%).

It is worth pointing out that in this CPG entrainment technique those doublets deemed *exceptional* can be reproduced at will, in contrast to the studies by Piotrkiewicz, where the volunteers were not trained to evoke doublets (Piotrkiewicz et al., 2013).

In regard to the morphology of the *D8 doublets*, our conjecture is that the κ and τ waves are the result of a first and a second motor unit firing, respectively, whereas the κ*ρσ*-complex would be the result arising from some superposition between multiple first and second motor unit firings.

### 4.3. Heart Rate Variability vs. Bursting Rate Variability

Similar to the normal resting heart rate range from 60 to 100 beats per minute (Peterkova and Stremy, 2015), here the observed doublet return time rate is between 60 and 88 cycles per minute on average among all the volunteers, which indicates a possible connection between HRV and BRV.

The observed D8 doublets that are absent during muscle relaxation, mild voluntarily contractions of the trunk, and while the person is not being entrained (see left panel of Figure 2 of Jonckheere et al., 2010) have different time parameters as those observed in a clinical ECG. Table 3.1 in Clifford et al. (2006) shows that the typical P-wave, QRS-complex, and corrected QT-interval durations for a healthy male adult have normal values and limits of 110±20 ms, 100±20 ms, and 400±40 ms respectively. Thus, a typical cardiac PQRST-wave duration would span a total of 510±60 ms. Furthermore, Figure 3 of Peterkova and Stremy (2015) shows a textbook example of an ECG cycle in normal conditions with a total duration of ~570 ms.

In this sEMG phenomenon, the π*κρστ*-wave and the κ*ρσ*-complex span shorter durations of ~130 ms and ~60 ms resp., compared with the equivalent cardiac PQRST-wave and QRS-complex durations of 510±60 and 110±20 ms resp.

Besides the difference in total wavelength between the cardiac cycle and “doublets,” it is worth stressing that here variability does not appear to occur *within* the doublet but rather *in its return time*, as the π*κρστ* wave duration of ~130 ms appears to be prevailingly fixed among doublets. This is unlike HRV, where a considerable amount of variability occurs among waves within same cardiac cycles (e.g., QT prolongation, Postema and Wilde, 2014).

To further exemplify the difference between a pure ECG trace and the π*κρστ* wave found here in the sEMG traces, studies show that the return time distributions of R-waves in ECG recordings have been found Erlang in normal subjects, and a weighted average of Erlang with a second distribution (e.g., Weibull) in patients with arrhythmia (Ariaei et al., 2008, 2015).

### 4.4. Off-Line and On-Line Bursting Rate Variability for Biofeedback Applications

For biofeedback applications, our objective is to help quadriplegic patients recover some motor control by learning how to evoke more doublet oscillations with return time distribution deviating from Weibull toward normal mixtures.

Another objective is to conduct *on-line* assessments by means of implementing the complete *off-line* technique described in section 2.5 with real-time DWT (Jaber and Bicker, 2015). For real-time muscle performance evaluations, it would reinforce the training process to help increase the number of synchronized motor units, resulting in stronger muscle contractions (Semmler, 2002).

The proposed technique is not restricted to only paraspinal muscles as it may span the evaluation of the neuromuscular system to a broader extent—for instance, to assess rhythmic involuntary contractions such as tremors, or uterine contractions in pregnancy. In the former, it could provide feedback to therapies in the field. In the latter, it would monitor the return times of uterine EMG bursts to potentially warn for signs of imminent, false, or preterm labor (Lucovnik et al., 2011).

### 4.5. Physiological Issue Underlying BRV

Probably the most challenging physiological problem to be addressed in BRV is the determination of whether the observed doublets recorded as muscle electric activity is produced by a single motor neuron discharging two closely spaced MUAPs, or by two motor neurons each carrying closely time-spaced MUAPs. The decomposed EMG (dEMG) or related technology might give the answer.

## 5. Conclusions

The major contribution in this paper is the identification of a new neurophysiological phenomenon—the *Bursting Rate Variability* that bears some resemblance to *Heart Rate Variability*, but that still differs from it in several respects, mainly single vs. double discharge. BRV is based on recursively shifting the Daubechies 3 wavelet transform of the raw electromyographic signal to successively provide time-localization and waveform characterization of spiking events by optimizing the waveform matching of the raw signal and its 8-level sub-signal. This 8-level subsignal in the raw SEMG signal is here referred to as “D8 doublets” due to the *two* adjacent and relatively high detail coefficients that span the *entire* rhythmic spiking phenomenon.

The presence of such *D8 doublets* in the sEMG signal has been conjectured to reveal coordination of muscle masses at a distance to achieve a higher hierarchy level movement. The return time statistic of the *D8 doublets* developed here adds some quantitative insights to this observation, with transition from *Weibull* to *normal mixture* distribution a possible indication of a quadriplegic subject recovering some motor control.

Cardiology applications and a plausible connection with ECG remain to be assessed by including electrocardiogram monitoring to our protocol and recording sEMG simultaneously.

Finally, from a theoretical viewpoint, this research is related to the statistic of return time of a dynamical system to some subset of its state space. The more recent Generalized Extreme Value (GEV) theory, which proceeds from the statistic of the extreme value of an observable (e.g., a sEMG signal) rather than the return time of such events, could offer an alternative way to look at the same phenomenon.

## Author's Note

The techniques developed in this paper are covered by US Patent Application No. US2020/0305743 A1, “Rhythmic synchronization of motor neuron discharges and their burst rate variability”.

## Data Availability Statement

The raw data supporting the conclusions of this article will be made available by the authors, without undue reservation.

## Ethics Statement

The studies involving human participants were reviewed and approved by University of Southern California University Park Institutional Review Board. The patients/participants provided their written informed consent to participate in this study.

## Author Contributions

Software development and simulation studies were performed by RM, based on earlier theoretical work of EJ. sEMG data was collected by EJ, with IRB approval. Both authors contributed to the article and approved the submitted version.

## Funding

This research was partially funded by the EpiEnergetic Foundation.

## Conflict of Interest

The authors declare that the research was conducted in the absence of any commercial or financial relationships that could be construed as a potential conflict of interest.

## Publisher's Note

All claims expressed in this article are solely those of the authors and do not necessarily represent those of their affiliated organizations, or those of the publisher, the editors and the reviewers. Any product that may be evaluated in this article, or claim that may be made by its manufacturer, is not guaranteed or endorsed by the publisher.
