# Supplementary figures and images for: Bursting Rate Variability
Source: Front Physiol. 2021 Dec 2;12:724027. doi: 10.3389/fphys.2021.724027 (PMC8674618; doi:10.3389/fphys.2021.724027)

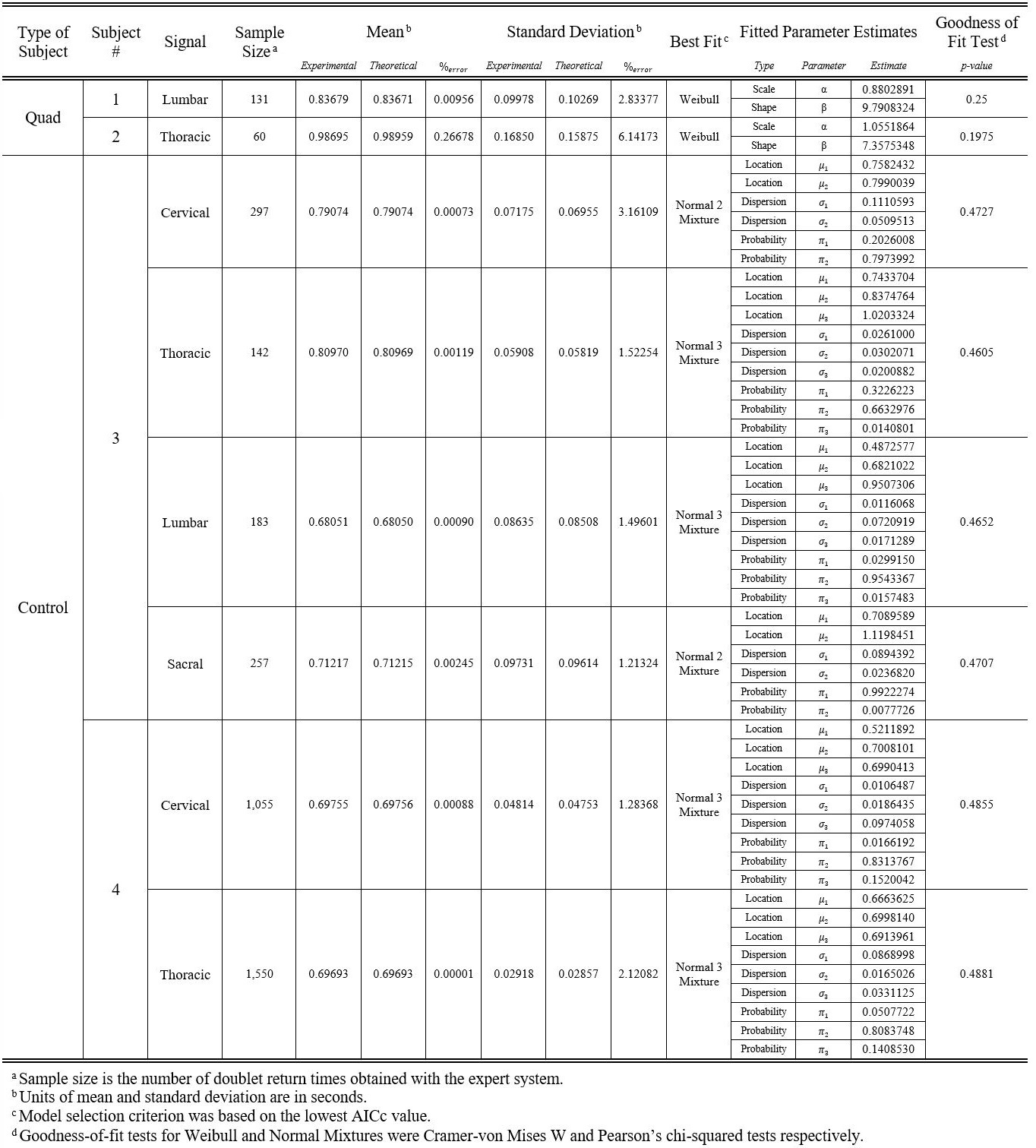

Supplement: Supplementary file 1 [file Data_Sheet_1.zip › Table1-1.JPG]

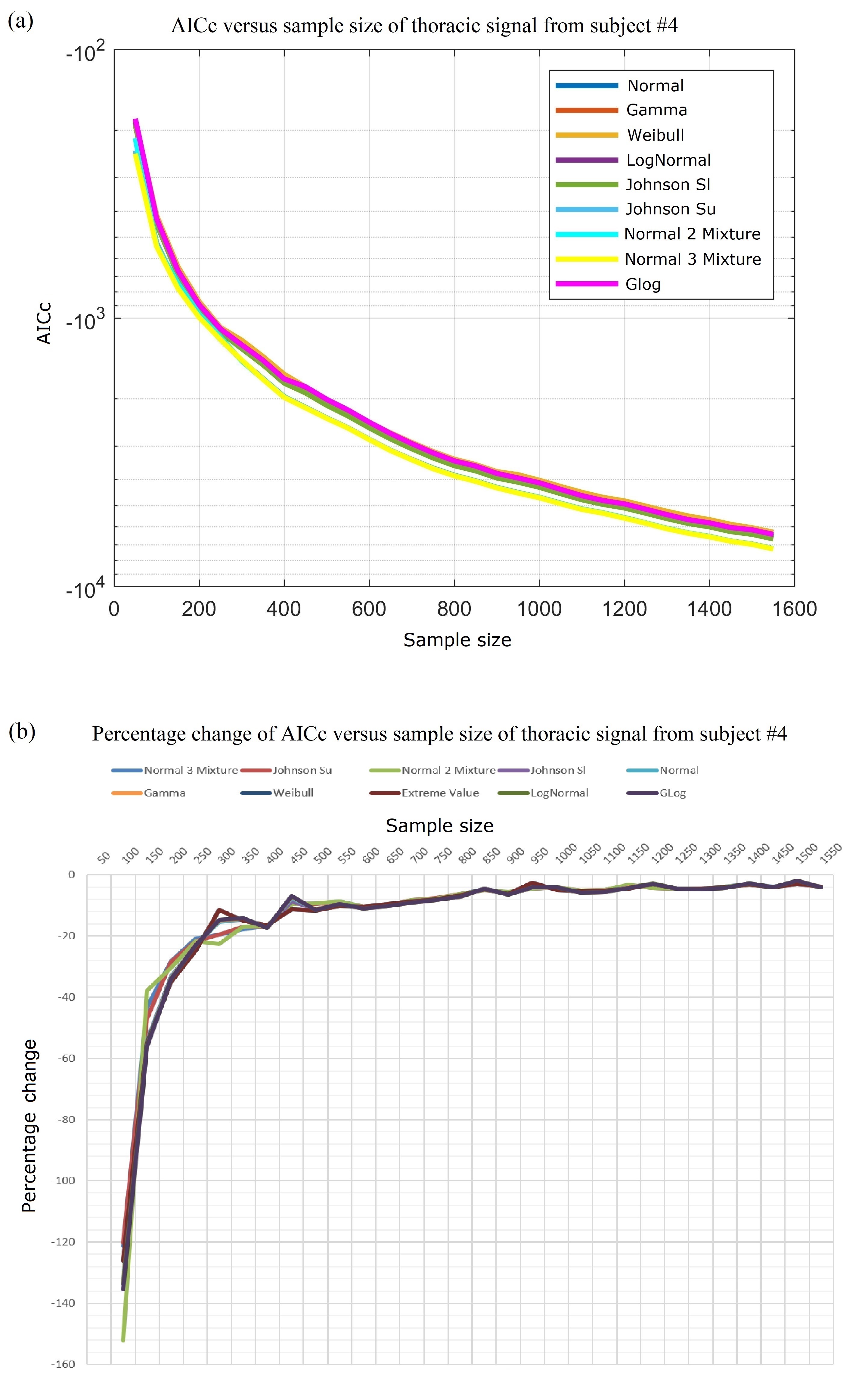

Supplement: Supplementary file 1 [file Data_Sheet_1.zip › FigureA3.jpg]

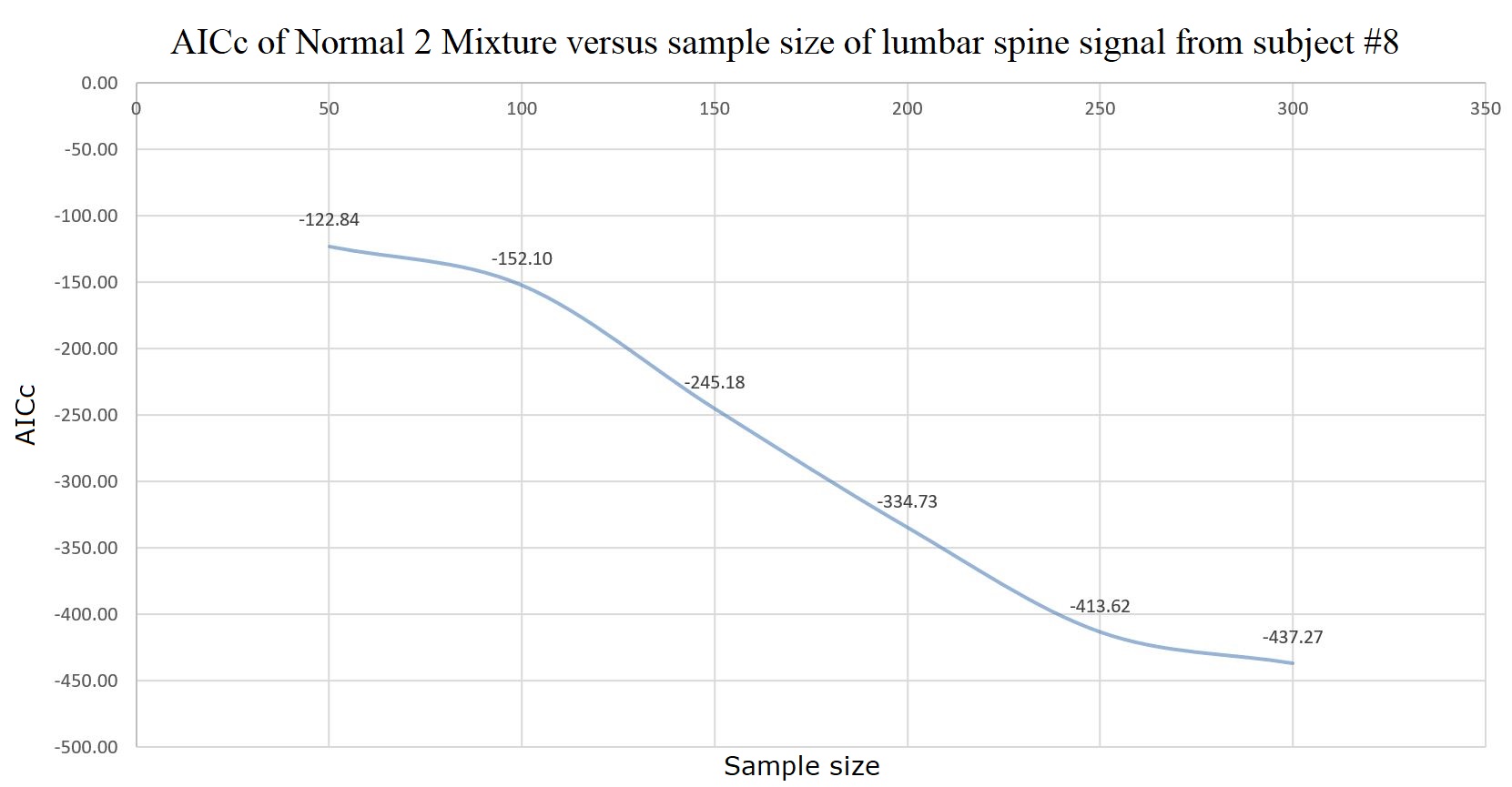

Supplement: Supplementary file 1 [file Data_Sheet_1.zip › FigureA4.JPG]

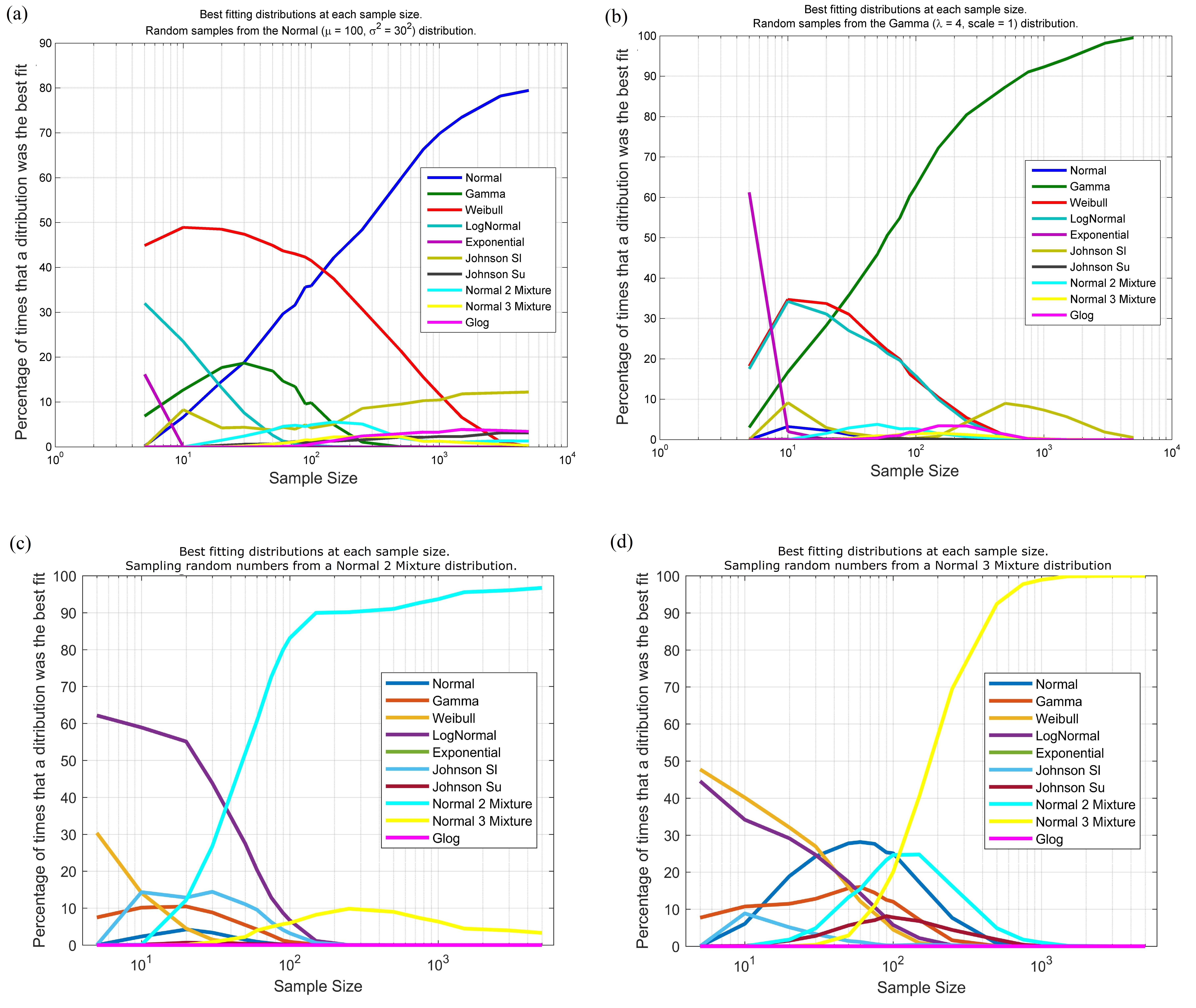

Supplement: Supplementary file 1 [file Data_Sheet_1.zip › FigureA2.jpg]

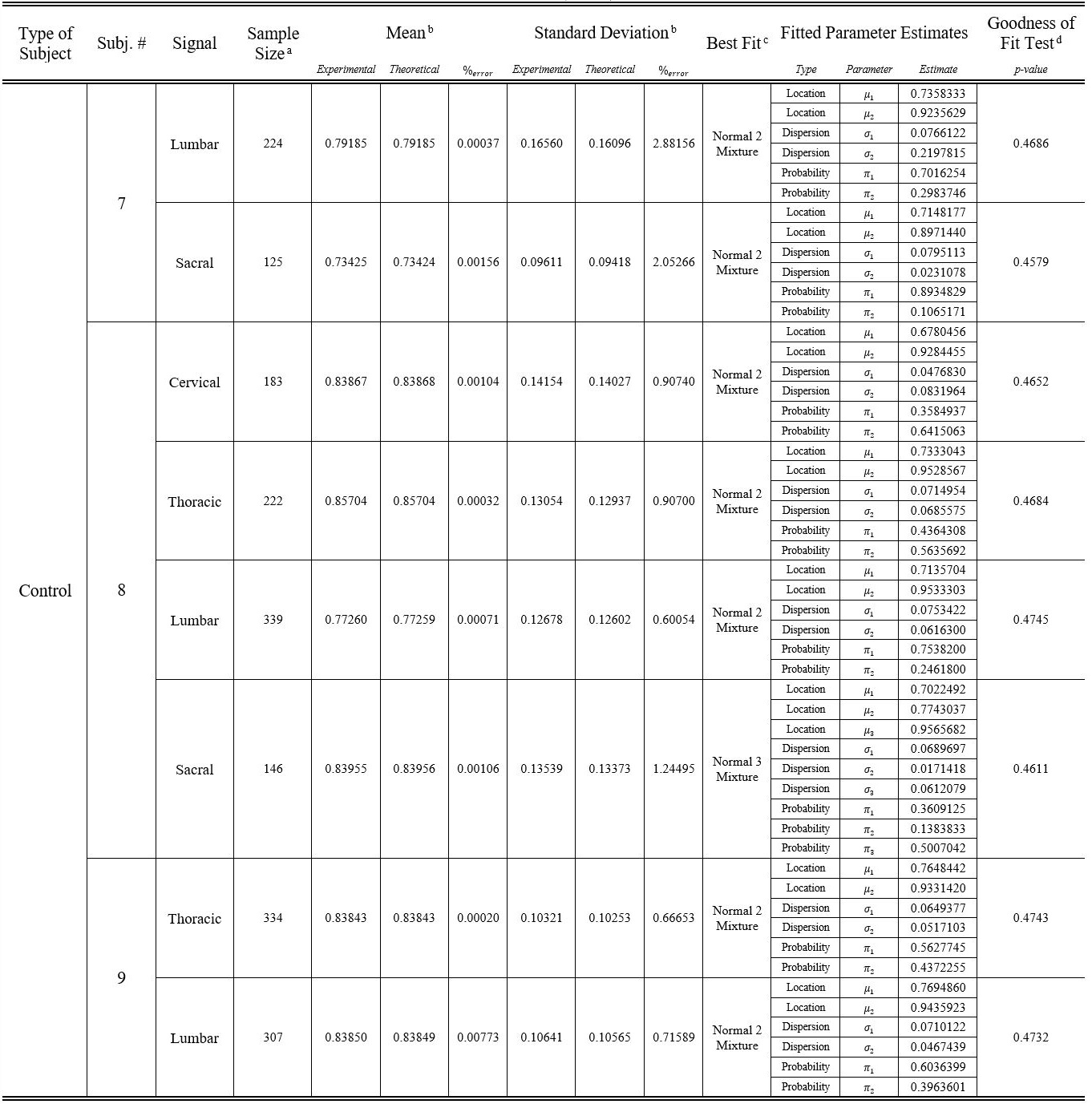

Supplement: Supplementary file 1 [file Data_Sheet_1.zip › Table1-3.JPG]

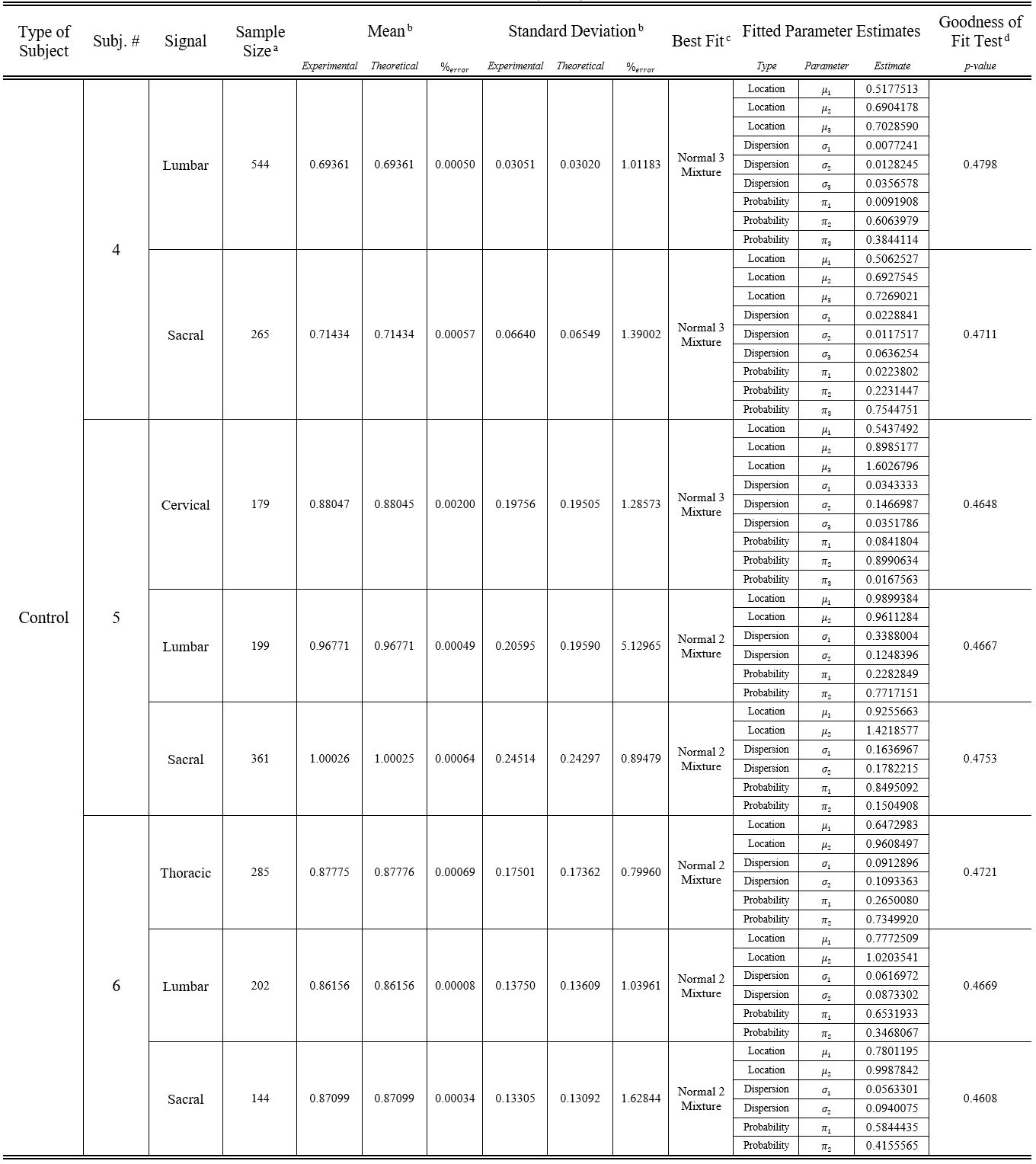

Supplement: Supplementary file 1 [file Data_Sheet_1.zip › Table1-2.JPG]

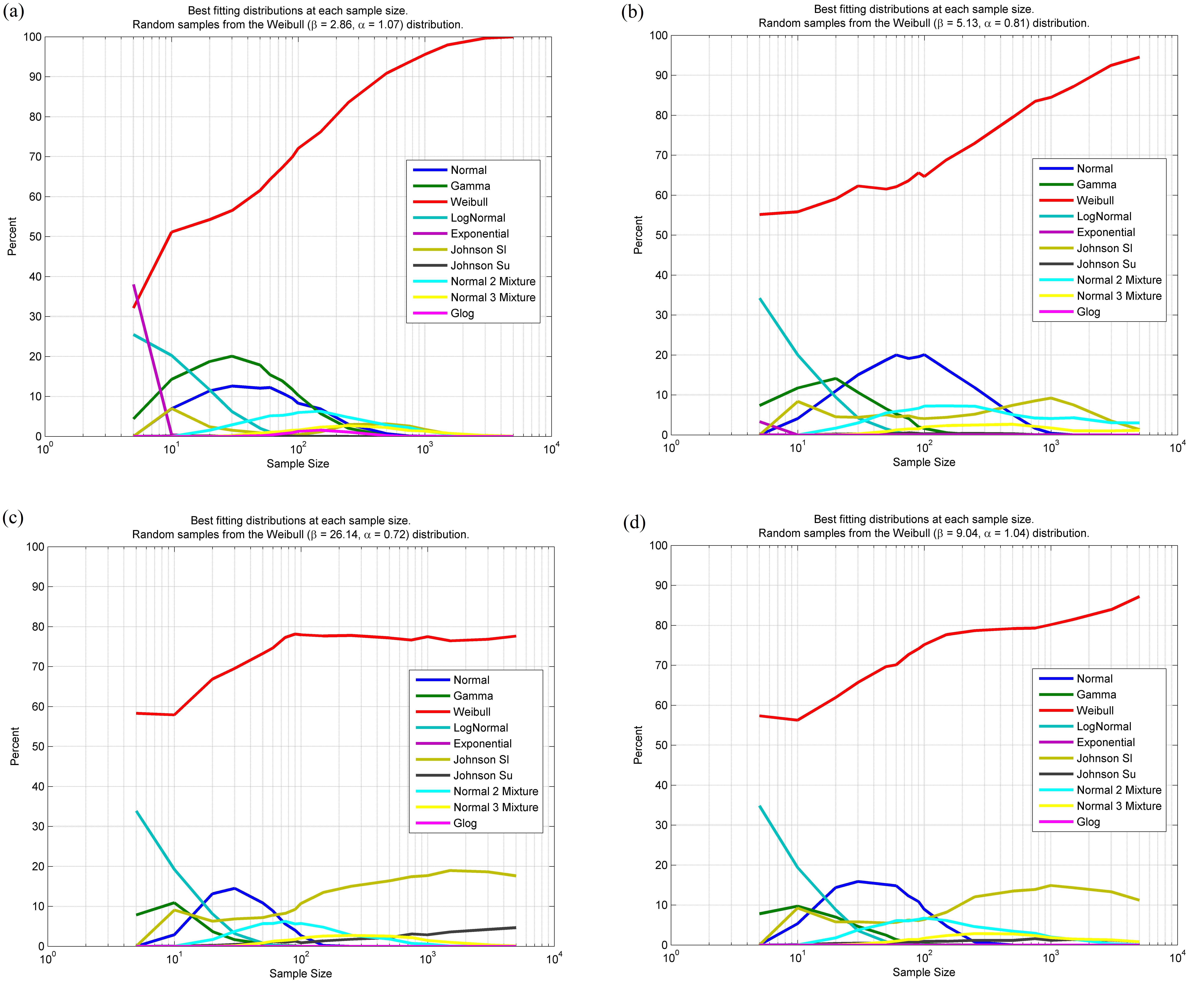

Supplement: Supplementary file 1 [file Data_Sheet_1.zip › FigureA1.jpg]
